# Supplementary material for: Antibody Profile, Gene Expression and Serum Cytokines in At-Risk Infants before the Onset of Celiac Disease
Source: Int J Mol Sci. 2023 Apr 6;24(7):6836. doi: 10.3390/ijms24076836 (PMC10095049; doi:10.3390/ijms24076836)
Supplement: Supplementary file 1 [file ijms-24-06836-s001.zip › ijms-1839481-supplementary.pdf]

SUPPLEMENTARY MATERIALS:

SUPPLEMENTARY TABLES:

TABLE S1. Means values of AGA and anti-tTG in celiac children

Age Months	AGA Mean	Low C.I.	Hi C.I.	Anti-tTG Mean	Low C.I.	Hi C.I.
4	1.27	0.99	1.62	1.00	1.00	1.00
6	2.17	1.52	3.10	1.00	1.00	1.00
9	2.96	1.89	4.65	0.12	0.10	0.13
12	3.54	2.24	5.60	0.12	0.11	0.13
16	7.71	1.07	55.77	0.34	0.01	12.81
18	4.29	2.45	7.54	0.39	0.16	0.95
22	9.12	1.64	50.69	2.27	0.16	31.49
24	4.50	2.45	8.28	0.81	0.27	2.43
26	4.09	0.79	21.07	8.37	0.57	122.82
30	4.58	2.22	9.47	2.54	0.24	26.40
36	5.12	2.60	10.07	3.26	0.98	10.88
42	38.77	2.11	158.49	27.15	0.79	120.23
48	4.66	2.43	8.92	1.06	0.30	3.77
60	4.69	1.67	13.18	2.66	0.48	14.61

TABLE S2. Correlation among serum antibodies in CeD and AGA+CTRLs

Correlation among antibodies in CeD				
		**anti-tTG IgA**	**DGP-IgA**	**DGP-IgG**
**AGA IgA**	r*	0.711	0.365	0.626
	p**	0.001	0.009	0.001
	n***	221	50	50
**Anti-tTG IgA**	r		0.669	0.807
	p		0.001	0.001
	n		49	49
**Correlation among antibodies in AGA+CTRLs**				
		**anti-tTG**	**DGP-IgA**	**DGP-IgG**
**AGA IgA**	r	-0.02	0.143	0.219
	p	0.769	0.342	0.154
	n	216	46	44

*r = Pearson correlation coefficient, **p = first degree error, ***n = sample size

TABLE S3. Differences in serum cytokines between PRE-CeD and CTRLs

		N	Mean	S.D.	S.E.M	Student t	p value
**LgINFγ***	CTRLs	26	0.1969	0.12281	0.02409		
	Pre-CeD	28	0.3944	0.32452	0.06133	-2.998	0.005
**LgIL1β**	CTRLs	26	0.1098	0.15338	0.03008		
	Pre-CeD	28	0.2353	0.26286	0.04968	-2.161	0.036
**LgIL2**	CTRLs	26	0.1148	0.14664	0.02876		
	Pre-CeD	28	0.4250	0.49919	0.09434	-3.145	0.004
**LgIL4**	CTRLs	26	0.1792	0.22145	0.04343		
	Pre-CeD	28	0.2909	0.38481	0.07272		
**LgIL6**	CTRLs	26	0.3253	0.29150	0.05717		
	Pre-CeD	28	0.5867	0.47191	0.08918	-2.468	0.017
**LgIL10**	CTRLs	26	0.1180	0.10683	0.02095		
	Pre-CeD	28	0.2453	0.28492	0.05385	-2.202	0.034
**LgIL12**	CTRLs	26	0.1772	0.03565	0.00699		
	Pre-CeD	28	0.2943	0.21757	0.04112	-2.808	0.009
**LgIL17A**	CTRLs	26	0.1525	0.11144	0.02186		
	Pre-CeD	28	0.3299	0.38455	0.07267	-2.338	0.026
**LgTNFα**	CTRLs	26	0.2146	0.21339	0.04185		
	Pre-CeD	27	0.3972	0.52561	0.10115		

* Log10 transformed cytokines in CTRLs and Pre-CeD

TABLE S4. Cytokine serum levels in non-celiac controls stratified on the base of AGA production

Group		LgINFγ	LgIL1β	LgIL2	LgIL4	LgIL6	LgIL10	LgIL12	LgIL17A	LgTNFα
CTRLs	Mean*	0.1969	0.1098	0.1148	0.1792	0.3253	0.1180	0.1772	0.1525	0.2146
	n	26	26	26	26	26	26	26	26	26
	SD	0.12281	0.15338	0.14664	0.22145	0.29150	0.10683	0.03565	0.11144	0.21339
AGA+CTRLs	Mean	0.2257	0.1390	0.1893	0.1892	0.3251	0.1010	0.2143	0.1997	0.2462
	n	17	17	17	17	17	17	17	17	17
	SD	0.16635	0.14316	0.26964	0.24610	0.23939	0.08594	0.13271	0.19146	0.29197

*Means and SD of log10 serum cytokines in controls according to the production of AGA

SUPPLEMENTARY FIGURES:

A

CeD

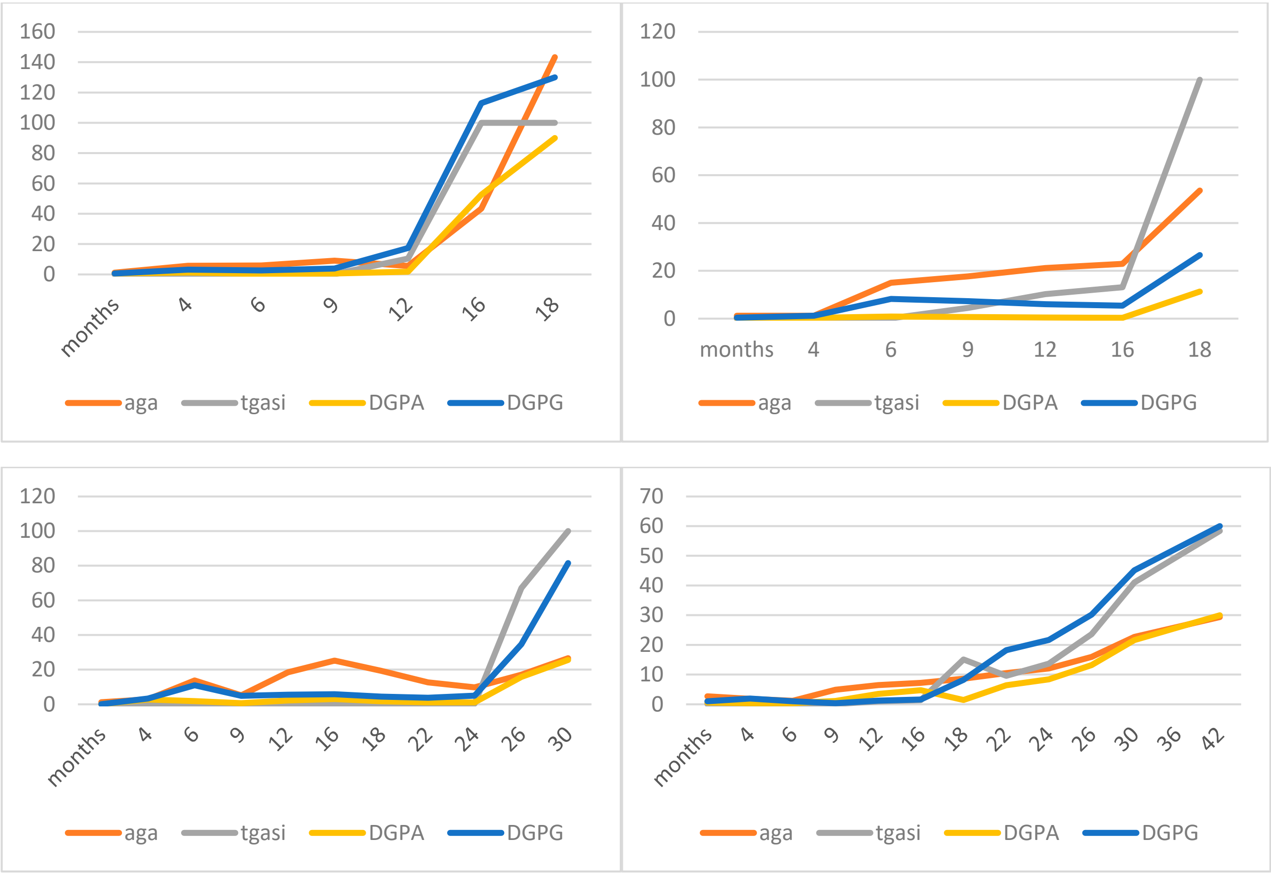

B

CTRLs

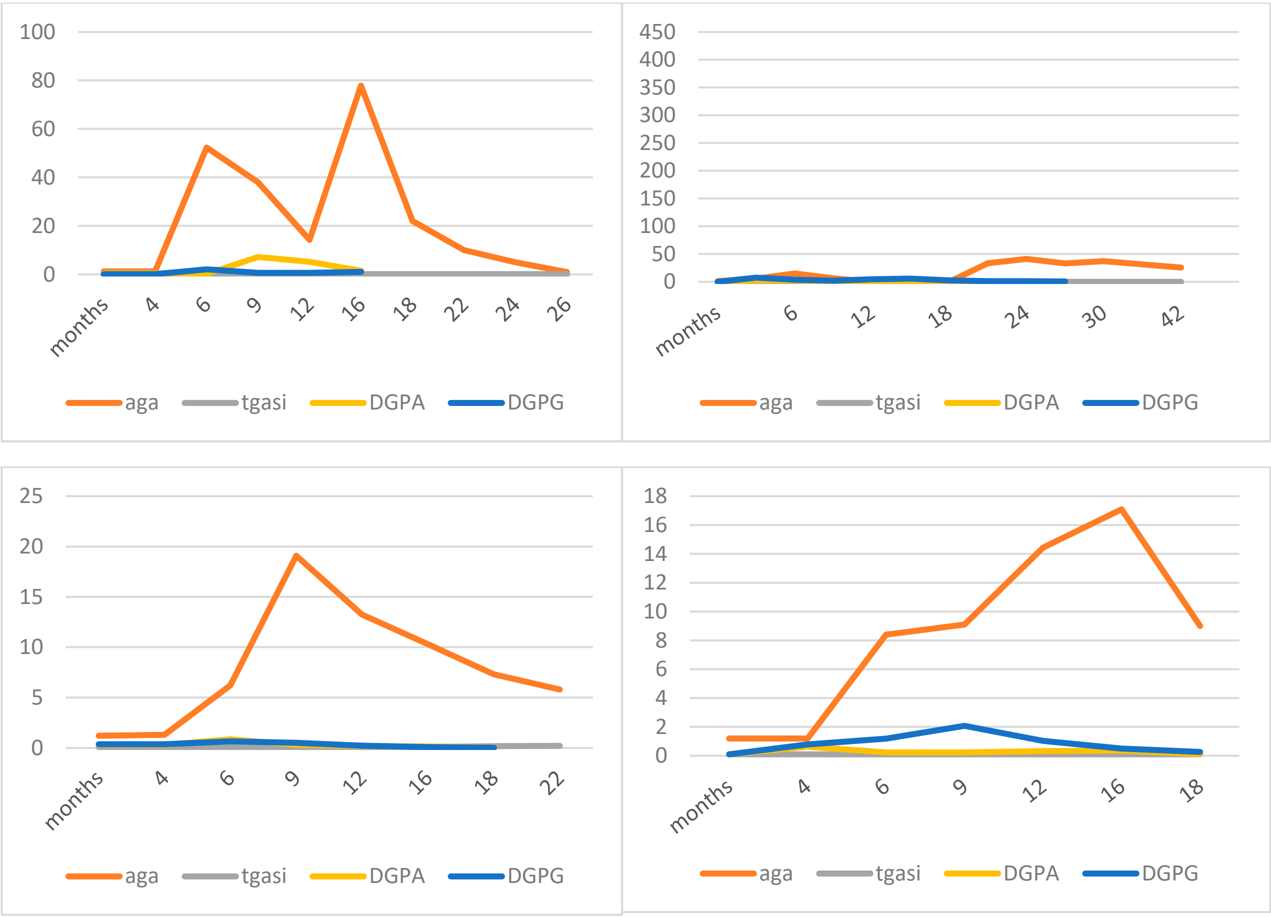

Figure S1. Individual profiles of antibodies in celiac children and healthy controls.

Profile of antibodies (AGA, anti-tTG IgA, DGP-IgA, DGP-IgG) of 4 representative celiac children (CeD, Panels A) and 4 healthy controls (CTRLs, Panels B).
